# Bilateral dystonia in type 1 diabetes: a case report

**DOI:** 10.1186/1752-1947-2-352

**Published:** 2008-11-18

**Authors:** Akihiro Yasuhara, Jun Wada, Hirofumi Makino

**Affiliations:** 1 Department of Medicine and Clinical Science, Okayama University Graduate School of Medicine 2-5-1, Shikata-cho, Okayama 700-8558, Japan

## Abstract

**Introduction:**

Diabetic hemichorea-hemiballismus is a rare complication of type 2 diabetes. Here, we report a case with type 1 diabetes, with hemichorea and bilateral dystonia manifested as hyperglycemia-induced involuntary movement.

**Case presentation:**

A 62-year-old Japanese women with body weight loss of 30 kg during the past year developed symptoms of thirst, polydipsia and polyuria. She also presented with hemichorea and bilateral dystonia for 5 days and extremely high plasma glucose (774 mg/dl), hemoglobin A1c (21.2%) and glycated albumin (100%) with ketosis. Based on the presence of glutamic acid decarboxylase antibodies (18,000 U/ml; normal <1.3 U/ml), low daily urinary excretion of C-peptide (7.8 μg), ketosis and human leucocyte antigen typing DR-4, we diagnosed type 1 diabetes mellitus. We treated the patient with a continuous intravenous regular insulin infusion and medication with haloperidol, and dystonia completely disappeared within 3 days.

**Conclusion:**

Hyperglycemia-induced involuntary movement is one of the manifestations of dystonia and hemichorea-hemiballism.

## Introduction

Chorea is defined as irregular, unpredictable, brief and jerky involuntary movements, while ballismus is large-amplitude flailing movements [1]. Hemichorea-hemiballismus is a rare complication of non-ketotic hyperglycemia and only 53 case reports of this particular condition were published between 1985 and 2001 [2]. Most of the cases were over 60 years of age and represented type 2 diabetes and non-ketotic hyperglycemia. The differential diagnosis of diabetic hemichorea-hemiballismus is challenging because this type of hyperkinetic movement disorder is caused by focal lesions, such as ischemic or hemorrhagic stroke, infection, epilepsy, and neoplasm, as well as systemic processes, including systemic lupus erythematosus, Wilson's disease and thyrotoxicosis [1]. Here, we present a case with type 1 diabetes dystonia manifesting ashyperglycemia-induced involuntary movement.

## Case presentation

A 62-year-old Japanese women with body weight loss of 30 kg during the past year developed symptoms of thirst, polydipsia and polyuria and was admitted to our hospital. She also presented with hemichorea and bilateral dystonia for 5 days. She forefelt onset for several seconds before initiation of involuntary movement. At first, she had chorea movement of her right arm at ~3 Hz, and then involuntarily and slowly elevated her right arm accompanied by continuing chorea movement of her right hand; she simultaneously stretched her right leg. About 10 seconds later, she slowly flexed her left knee and maintained a bilateral and asymmetrical spastic posture. The sequences of slow and continuous muscular contractive movement were defined as bilateral "dystonic movement". The whole series of movements terminated in 30 seconds and she was finally relieved from her dystonia and could voluntarily move again (see Additional file 1). Since exactly the same pattern of hemichorea and bilateral dystonic movement occurred intermittently every 10 minutes, she could not stand and had had difficulties in taking meals for 2 days. These movements were observed in both waking and sleep states. She presented extremely high plasma glucose (774 mg/dl), hemoglobin A1c (21.2%) and glycated albumin (100%) with ketosis but without acidosis. Anti-nuclear antibodies were negative, and serum ceruloplasmin and thyroxine levels were within the normal range. Magnetic resonance imaging (MRI) demonstrated no brain tumor, hemorrhage and infarction and she had a normal electroencephalogram excluding the possibility of epilepsy. MR images were not typical of diabetic hemichorea-hemiballismus which would show high signal basal ganglia lesions, mainly putamen, on T1-weighted images [3]. Based on the presence of glutamic acid decarboxylase antibodies (18,000 U/ml; normal <1.3 U/ml), low daily urinary excretion of C-peptide (7.8 μg), ketosis and human leucocyte antigen (HLA) typing DR-4, we diagnosed type 1 diabetes mellitus. We treated the patient with continuous intravenous regular insulin infusion and medication with haloperidol, and dystonia completely disappeared within 3 days. After the discontinuation of haloperidol, recurrence of dystonia was not observed.

## Discussion

Many hypotheses have been reported for the development of diabetic hemichorea-hemiballismus, such as local gamma-aminobutyric acid (GABA) starvation, disinhibition of dopaminergic neurons, local microhemorrhage, microinfarction, demyelination and brain edema [4]. Recent imaging analysis has revealed reduced cerebral glucose metabolism on positron emission tomography (PET) scans with concomitant hyperperfusion in affected basal ganglia seen on single photon emission computed tomography (SPECT) [5]. In some cases, the basal ganglia in diabetic hemichorea-hemiballismus were hyperdense without mass effect on computed tomography (CT) scans and hyperintense on T1-weighted magnetic resonance imaging (MRI) scans but these imaging features completely reversed after therapy [2]. This evidence supports the idea that basal ganglia are generally weak in hyperglycemic stress, and chronic hyperglycemic stress might induce reversible neurotransmitting functional disorders and consequent involuntary movement. Since dystonia is caused by lesions of the basal ganglia, it is a spectrum of hyperglycemia-induced involuntary movements in addition to hemichorea-hemiballism.

Diabetic hemichorea-hemiballismus is mostly observed in type 2 diabetes and cases with type 1 diabetes are extremely rare. In the 53 cases reported in the literature, only one case of type 1 diabetes with acute onset of non-ketotic hyperglycemia was reported and the rest were type 2 diabetes in elderly patients [2]. This case series suggests that long-term exposure to hyperglycemia without ketosis in the elderly is related to the development of hemichorea-hemiballismus in diabetes. We speculate that our patient was exposed to long-term hyperglycemic stress because she manifested a slowly progressive form of type 1 diabetes without acidosis states.

## Conclusion

Hyperglycemia-induced involuntary movement is one of the manifestations of dystonia and hemichorea-hemiballism.

## Consent

Written informed consent was obtained from the patient for publication of this case report and any accompanying images. A copy of the written consent is available for review by the Editor-in-Chief of this journal.

## Competing interests

The authors declare that they have no competing interests.

## Authors' contributions

HM analyzed and interpreted the data regarding type I diabetes and MRI imaging. AY and JW contributed to study concept and design, patient care, data analysis, literature review, and writing the manuscript. All authors read and approved the final manuscript.

## Supplementary Material

Additional file 1 **Hemichorea and bilateral dystonia in our patient.** The complete series of movements terminated in 30 seconds. Exactly the same pattern of hemichorea and bilateral dystonic movement occurred intermittently every 10 minutes. Click here for file
